# At what level of unconsciousness is mild therapeutic hypothermia indicated for out-of-hospital cardiac arrest: a retrospective, historical cohort study

**DOI:** 10.1186/s40560-015-0104-5

**Published:** 2015-09-11

**Authors:** Tomoaki Natsukawa, Hirotaka Sawano, Mai Natsukawa, Yuichi Yoshinaga, Shuho Sato, Yusuke Ito, Takayuki Otani, Jiro Ohba, Yasuyuki Hayashi, Tatsuro Kai

**Affiliations:** Senri Critical Care Medical Center, Osaka Saiseikai Senri Hospital, 1-1-6 Tsukumodai, Suita City, Osaka Japan

**Keywords:** Mild therapeutic hypothermia, Post cardiac arrest, Coma, Glasgow Coma Scale, Motor response score

## Abstract

**Background:**

Appropriate patient selection is very important when initiating mild therapeutic hypothermia (MTH) for patients following out-of-hospital cardiac arrest, and the extent of unconsciousness at implementation must be defined in such cases. However, there are no clear standards regarding the level of unconsciousness at which MTH would be beneficial. The effects of MTH in patients with different degrees of unconsciousness according to the motor response score of the Glasgow Coma Scale (GCS) were investigated.

**Methods:**

The subjects consisted of witnessed non-traumatic adult out-of-hospital cardiac arrest patients admitted to our institute from April 2002 to August 2011. The patients were divided into six groups according to the GCS motor response score: 1 (GCS M1), 2 (GCS M2), 3 (GCS M3), 4 (GCS M4), 5 (GCS M5), and 6 (GCS M6). The neurological outcome was evaluated at 30 days after hospital admission using the Cerebral Performance Category. Chi-squared Automatic Interaction Detection (CHAID) analysis was performed to estimate the threshold GCS M level where therapeutic hypothermia is indicated. Odds ratios were then calculated by multiple logistic-regression analysis using factors including GCS M5–6 and MTH.

**Results:**

A total of 289 patients were enrolled in this study. CHAID analysis demonstrated two points of significant increase in percentage of good recovery at 30 days after admission, dividing the GCS M categories into three groups. Patients classified with a GCS motor response score of 5 or higher had the highest percentage of good recovery. The odds ratio for good recovery (CPC1–2) was 2.901 (95 % CI 1.460–5.763, *P* = 0.002) for MTH, and that for GCS M5–6 was 159.835 (95 % CI 33.592–760.513, *P* < 0.001).

**Conclusions:**

MTH may be unnecessary in patients with a GCS motor response score of 5 or higher. Consequently, because there are post cardiac arrest patients with a GCS motor response score of 4 or lower who benefit from MTH, MTH may be limited to patients with a GCS motor response score of 4 or lower.

## Background

The American Heart Association currently recommends mild therapeutic hypothermia (MTH) for out-of-hospital cardiac arrest patients presenting in a coma [1].

MTH is a therapy capable of improving the neurological outcome of post cardiac arrest patients, but it is also associated with many complications [2]. MTH, which includes precise body temperature management, is a therapy that requires an increase in manpower in the intensive care unit (ICU), thus creating problems due to increased medical costs, including the purchase of equipment and special devices. Therefore, the indications to perform MTH in patients with out-of-hospital cardiac arrest should be examined.

The Glasgow Coma Scale (GCS) is generally used for evaluating the state of consciousness at emergency scenes [3]. There is no definition of the degree of unconsciousness for which MTH is indicated at the present time, although in past reports, a GCS of eight points or less has been reported. However, it is difficult to evaluate eye opening when the patient is in a post cardiac arrest coma. Moreover, tracheal intubation is carried out in almost all cases, thus rendering an evaluation of the verbal response impossible. From the above, when evaluating the state of consciousness in cardiac arrest patients using the GCS, only the motor response is commonly used. Regarding the evaluation of the degree of unconsciousness in out-of-hospital cardiac arrest patients on arrival to the hospital, we have conventionally used the best level of consciousness within 2 h following the return of circulation, before administration of sedatives, using only the GCS motor response score. Patients classified with a GCS motor response score of 3 or lower are considered to be in a comatose state. Meanwhile, although patients classified with a GCS motor response score of 4 or higher may or may not be in a comatose state, it is believed that MTH may have a positive effect on patient outcome.

In this study, the level of consciousness of out-of-hospital cardiac arrest patients on arrival to the hospital was classified according to the GCS motor response score, and the effect of MTH was retrospectively investigated. In addition to identifying the indications for MTH using the GCS motor response score, whether classifying such patients according to their GCS motor response score on arrival to the hospital may be a potentially useful indicator for predicting the outcome was investigated.

## Methods

### Subjects

The subjects were adults who were 18 years of age or older who had been admitted to our institute due to witnessed out-of-hospital cardiac arrest from April 2002 to August 2011 with stable post cardiac arrest hemodynamics. Patients with unwitnessed cardiac arrest or patients with injury, cerebral stroke, a poor functional status, in a terminal state, a serious infectious disease, and serious coagulopathy were excluded. There was no clear criterion for the use of MTH in our institute. MTH was conducted when two or more emergency physicians who were in charge of the treatment thought that MTH would be beneficial.

### Methods

The patients’ data were extracted from medical records, and the data, as well as the outcomes, were retrospectively analyzed as a historical cohort study. The best level of consciousness within 2 h following return of spontaneous circulation (ROSC) was evaluated using the GCS motor response score. Once a decision was made to perform MTH, the administration of a rapid intravenous infusion of 2000 ml of cooling liquid at 4 °C was carried out immediately, along with commencing body surface cooling in most patients. The core body temperature was continuously decreased to 34 °C using the K-TEK III® (Kawasumi, Tokyo), ArcticSun® (IMI, Saitama), or percutaneous cardio-pulmonary support (PCPS), and the core body temperature was then maintained at 34 °C for 24 h, before rewarming over a period of 2 days at 1 °C/day. Furthermore, sedatives, analgesics, and muscle relaxant agents were used in all patients during MTH, and the bladder temperature was monitored as the core body temperature. Meanwhile, sedatives and analgesics were used in patients who were treated without MTH, and although monitoring of the core body temperature was carried out, no active intervention was carried out for temperature management. The outcome, the state of consciousness at 30 days of hospital admission, was evaluated using the Cerebral Performance Category (CPC) [4]. CPC1 and CPC2 were determined to be a good recovery.

The study protocol complied with the guidelines for epidemiologic studies issued by the Ministry of Health, Labour and Welfare of Japan and was approved by the Ethics Committee of the Osaka Saiseikai Senri Hospital. All patients received the standard care available at the hospital, and no subjects underwent any type of experimental intervention. In light of these safeguards, the Ethics Committee approved this study and waived the need for oral or written consent.

### Statistical analyses

Continuous variables, which were not normally distributed, are reported as medians and interquartile ranges. Categorical variables are reported as counts and percentages. Either the chi-square test or Fisher’s exact test, as appropriate, was used to compare neurological outcomes in patients treated and those not treated with MTH. The Mann-Whitney *U* test was used to compare continuous variables of baseline characteristics between patients with good recovery (CPC1–2) and those with bad recovery (CPC3–5). Chi-squared Automatic Interaction Detection (CHAID) analysis was used to find the threshold of GCS M in patients with good recovery at 30 days after admission. Odds ratios (ORs) were calculated by multivariate logistic-regression analysis to identify the significant factors for good recovery. The significance level was set at *P* < 0.05. The software program SPSS Statistics 21 for Windows (IBM Japan, Tokyo) was used to analyze the data.

## Results

### Patient selection

**Fig. 1:**
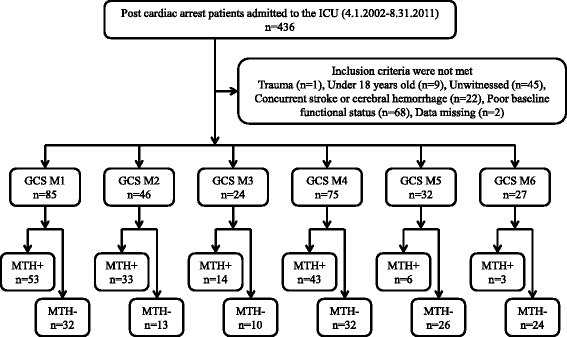
Flow chart of patient selection. *ICU* intensive care unit, *GCS M1* patients classified with a GCS motor response score of 1, *GCS M2* patients classified with a GCS motor response score of 2, *GCS M3* patients classified with a GCS motor response score of 3, *GCS M4* patients classified with a GCS motor response score of 4, *GCS M5* patients classified with a GCS motor response score of 5, *GCS M6* patients classified with a GCS motor response score of 6, *MTH+* patients who were treated with mild therapeutic hypothermia, *MTH*− patients who were treated without mild therapeutic hypothermia

A total of 436 out-of-hospital cardiac arrest patients were admitted to our institute from April 2002 to August 2011. One patient with trauma, nine patients under 18 years of age, 45 unwitnessed patients, 22 patients with cerebral stroke, 68 patients with poor functional status, and two patients with faulty data were excluded, thus leaving 289 patients enrolled in this study (Fig. 1).

### Baseline characteristics

The background characteristics of all patients are shown in Table 1. There was a significant bias in the distribution of patients with good recovery by GCS score (*P* < 0.001). The percentage of patients with a good recovery was 10.6 % in the GCS M1 group, 41.3 % in the GCS M2 group, 50.0 % in the GCS M3 group, 56.0 % in the GCS M4 group, 96.9 % in the GCS M5 group, and 92.6 % in the GCS M6 group. No significant difference was observed in the percentage receiving MTH between patients with and without a good recovery (*P* = 0.795).

**Table 1:** Baseline clinical characteristics

Parameters	Good recovery	Bad recovery	*P* value
	CPC 1–2 (*n* = 138)	CPC 3–5 (*n* = 151)	
Age (year), median (IQR)	60 (53, 73)	66 (58, 73)	<0.001
Male, *n*/total *n* (%)	110/138 (79.7)	114/151 (75.5)	0.392
Bystander CPR, *n*/total *n* (%)	79/138 (57.2)	66/151 (43.7)	0.022
VF or pulseless VT, *n*/total *n* (%)	65/138 (47.1)	54/151 (35.8)	0.050
Cardiac cause, *n*/total *n* (%)	67/138 (48.6)	50/151 (33.1)	0.007
Time interval between collapse and ROSC (min), median (IQR)	14 (10, 22)	25 (18, 42)	<0.001
Without prehospital ROSC, *n*/total *n* (%)	12/138 (8.7)	40/151 (26.5)	<0.001
Glasgow Coma Scale motor response score			
1	9	76	
2	19	27	
3	12	12	<0.001
4	42	33	
5	31	1	
6	25	2	
Laboratory Data			
pH, median (IQR)	7.27 (7.19, 7.34)	7.09 (6.94, 7.26)	<0.001
Base excess (mmol/l), median (IQR)	−7.8 (−14.1, −4.9)	−13.7 (−18.7, −10.4)	<0.001
Lactate (mmol/l), median (IQR)	6.8 (4.8, 9.4)	9.8 (7.3, 12.1)	<0.001
PCPS, *n*/total *n* (%)	11/138 (8.0)	44/151 (29.1)	< 0.001
IABP, *n*/total *n* (%)	12/138 (8.7)	44/151 (29.1)	<0.001
MTH	71/138 (51.4)	80/151 (53.0)	0.795

*IQR* interquartile range, *CPR* cardio-pulmonary resuscitation, *VF* ventricular fibrillation, *VT* ventricular tachycardia, *ROSC* return of spontaneous circulation, *PCPS* percutaneous cardio-pulmonary support, *IABP* intra-aortic balloon pumping, *MTH* mild therapeutic hypothermia

The median (IQR) time interval between ROSC and consciousness assessment in each GCS M group was as follows: GCS M1, 66 (36, 89); GCS M2, 48 (33, 77); GCS M3, 52 (36, 80); GCS M4, 49 (34, 83); GCS M5, 77 (32, 113); and GCS M6, 48 (20, 69).

### Neurological outcome

The CPCs at 30 days after hospital admission by MTH use are shown in Table 2. In the GCS M2 and GCS M4 groups, patients treated with MTH had a significantly better neurological outcome at 30 days after hospital admission (*P* = 0.006, *P* = 0.005). Meanwhile, in the GCS M1 group, GCS M3 group, GCS M5 group, and GCS M6 group, no significant difference was observed (*P* = 0.910, *P* = 0.558, *P* = 0.952, *P* = 0.542).

**Table 2:** Neurological outcome at 30 days after hospital admission by MTH

	CPC	1	2	3	4	5	*P* value
GCS M1	MTH+	6	1	5	19	22	0.910
	MTH−	1	1	2	13	15	
GCS M2	MTH+	14	5	1	9	4	0.006
	MTH−	0	0	6	6	1	
GCS M3	MTH+	5	3	0	3	3	0.558
	MTH−	1	3	3	2	1	
GCS M4	MTH+	25	5	3	6	4	0.047
	MTH−	6	6	5	6	9	
GCS M5	MTH+	5	1	0	0	0	0.952
	MTH−	24	1	0	0	1	
GCS M6	MTH+	2	0	0	0	1	0.542
	MTH−	23	0	1	0	0	

*CPC* Cerebral Performance Category, *MTH+* patients who were treated with mild therapeutic hypothermia, *MTH−* patients who were treated without mild therapeutic hypothermia, *GCS M1* patients classified with a GCS motor response score of 1, *GCS M2* patients classified with a GCS motor response score of 2, *GCS M3* patients classified with a GCS motor response score of 3, *GCS M4* patients classified with a GCS motor response score of 4, *GCS M5* patients classified with a GCS motor response score of 5, *GCS M6* patients classified with a GCS motor response score of 6

Furthermore, one GCS M5 case with CPC5 that had not undergone MTH was admitted to the hospital due to malnutrition and died in hospital due to an inability to control the primary disease. In this case, it was difficult to believe that MTH would have improved the CPC at 30 days after hospital admission. Moreover, one GCS M6 case with CPC5 that underwent MTH was a case in which death was caused without recovery due to pump failure due to acute myocardial infarction, and one case with CPC3 that had not undergone MTH was a case in which decreased activity was due to extended critical illness hospitalization caused by old age. Regarding these two cases as well, it was difficult to believe that implementing MTH would have improved the CPC at 30 days after hospital admission.

**Fig. 2:**
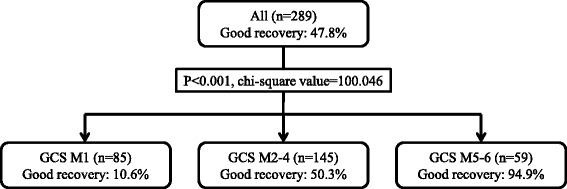
Chi-squared Automatic Interaction Detection classification tree for good recovery at 30 days after hospital admission. *GCS M1* patients classified with a GCS motor response score of 1, *GCS M2–4* patients classified with a GCS motor response score from 2 to 4, *GCS M5–6* patients classified with a GCS motor response score of 5 or higher

CHAID analysis was performed with GCS M as an independent variable and good recovery at 30 days after admission as a dependent variable. The tree created after applying CHAID is shown in Fig. 2. The terminal branches of the tree represent CHAID-derived homogeneous categories (terminal nodes). We obtained three terminal nodes. Regarding GCS M, there were two points of significant increase in percentage of good recovery at 30 days after admission, dividing the GCS M categories into three groups (see Fig. 2). Patients classified with a GCS motor response score of 5 or higher had the highest percentage of good recovery.

### Odds ratio for a good recovery

A multivariate logistic-regression analysis was performed for all patients, and ORs were calculated to identify significant factors associated with a good recovery (Table 3). In multivariate 1, a multivariate logistic-regression analysis was performed, and the ORs were calculated using all parameters. In multivariate 2, age, bystander CPR, ventricular fibrillation (VF) or pulseless ventricular tachycardia (VT), without prehospital ROSC, GCS M score 5–6, lactate, and MTH were used. As a rule, it is better not to perform multiple logistic-regression analysis including factors that are strongly correlated. Because pH, base excess, and lactate were strongly correlated with each other (pH vs. base excess: *r* = 0.856, *P* < 0.001; pH vs. lactate: *r* = −0.673, *P* < 0.001; base excess vs. lactate: *r* = −0.770, *P* < 0.001), only lactate was selected. In the same way, because without prehospital ROSC, PCPS, and IABP were strongly correlated with each other (without prehospital ROSC vs. PCPS: *r* = 0.599, *P* < 0.001; without prehospital ROSC vs. IABP: *r* = 0.522, *P* < 0.001; PCPS vs. IABP: *r* = 0.744, *P* < 0.001) and without prehospital ROSC and time interval between collapse and ROSC were strongly correlated with each other (*r* = 0.634, *P* < 0.001), only without prehospital ROSC was selected. Because VF or pulseless VT and cardiac cause are strongly correlated with each other (*r* = 0.509, *P* < 0.001), only VF or pulseless VT was selected.

**Table 3:** Odds ratio for a good recovery

Parameters	Good recovery (CPC 1–2)		
	Univariate	Multivariate 1	Multivariate 2
	Odds ratio (95 % CI)	Odds ratio (95 % CI)	Odds ratio (95 % CI)
Age (1 year)	0.969 (0.953–0.985)	0.955 (0.931–0.980)	0.958 (0.935–0.979)
Male	1.275 (0.731–2.224)	0.806 (0.355–1.831)	
Bystander CPR	1.724 (1.082–2.748)	2.064 (1.022–4.169)	1.813 (0.957–3.433)
VF or pulseless VT	2.040 (1.264–3.289)	1.505 (0.632–3.581)	2.492 (1.247–4.980)
Cardiac cause	2.123 (1.203–3.745)	2.800 (0.995–7.882)	
Time interval between collapse and ROSC (1 min)	0.949 (0.931–0.967)	0.972 (0.944–1.000)	
Without prehospital ROSC	0.264 (0.132–0.529)	0.521 (0.135–2.020)	0.330 (0.125–0.867)
GCS Motor response score 5–6	33.691 (10.225–111.015)	123.272 (26.842–566.123)	159.835 (33.592–760.513)
pH (0.1)	1.564 (1.352–1.809)	1.397 (0.981–1.988)	
Base Excess (1 mmol/l)	1.107 (1.066–1.150)	1.017 (0.911–1.137)	
Lactate (1 mmol/l)	0.834 (0.778–0.895)	0.914 (0.797–1.047)	0.830 (0.752–0.916)
PCPS	0.211 (0.104–0.428)	0.541 (0.125–2.342)	
IABP	0.232 (0.116–0.461)	0.298 (0.079–1.129)	
MTH	1.033 (0.651–1.640)	2.980 (1.414–6.281)	2.901 (1.460–5.763)

*CPR* cardio-pulmonary resuscitation, *VF* ventricular fibrillation, *VT* ventricular tachycardia, *ROSC* return of spontaneous circulation, *GCS M5-6* patients classified with a GCS motor response score of 5 or higher, *PCPS* percutaneous cardio-pulmonary support, *IABP* intra-aortic balloon pumping, *MTH* mild therapeutic hypothermia

In multivariate 1, significant factors associated with a good recovery were age (1 year) (OR 0.955, *P* < 0.001, 95 % confidence interval 0.931 to 0.980), bystander CPR (OR 2.064, *P* = 0.043, 95 % confidence interval 1.022 to 4.169), GCS M5–6 (OR 123.272, *P* < 0.001, 95 % confidence interval 26.842 to 566.123), and MTH (OR 2.980, *P* = 0.004, 95 % confidence interval 1.414 to 6.281).

In multivariate 2, significant factors associated with a good recovery were age (1 year) (OR 0.958, *P* < 0.001, 95 % confidence interval 0.934 to 0.979), VF or pulseless VT (OR 2.492, *P* = 0.010, 95 % confidence interval 1.247 to 4.980), without prehospital ROSC (OR 0.330, *P* = 0.025, 95 % confidence interval 0.125 to 0.867), GCS M5–6 (OR 159.835, *P* < 0.001, 95 % confidence interval 33.592 to 760.513), lactate (1 mmol/l) (OR 0.830, *P* < 0.001, 95 % confidence interval 0.752 to 0.916), and MTH (OR: 2.901, *P* = 0.002, 95 % confidence interval 1.460 to 5.763).

## Discussion

The GCS is a useful indicator for evaluating the level of consciousness in emergency situations, which is also suitable for evaluating the level of consciousness post cardiac arrest. Specifically, the motor response score reflects an abnormal reflex and integrated movement, thus reflecting the degree of encephalopathy. However, it is difficult to evaluate eye opening when the patient is in a post cardiac arrest coma. For example, we sometimes find post cardiac arrest patients who present with open eyes, but they cannot obey an order to close their eyes. Thus, they do not truly have an eye opening score of 4 in the GCS. In our opinion, it would be better not to take the eye opening score of the GCS into account. Moreover, tracheal intubation is carried out in almost all cases and even if using the modified verbal score, it would be difficult to evaluate the GCS verbal response score as usual. Therefore, the verbal response score is obscure in most post cardiac arrest patients. From the above, when evaluating the state of consciousness in cardiac arrest patients, it is believed that evaluating such patients using only the motor response is most appropriate.

The definitions regarding the degree of unconsciousness where MTH is indicated in past reports are shown in Table 4 [5–17]. There are no clear standards regarding the level of unconsciousness required for MTH in previous papers. Some previous papers on MTH for post cardiac arrest syndrome included GCS M5 patients. Others did not define coma. As for standards regarding the level of unconsciousness required for MTH, the American Heart Association guideline 2010 states the following. “Who to cool? All studies of post cardiac arrest therapeutic hypothermia have included only patients in coma. One trial defined coma as “not responding to verbal commands”. The other trials defined coma similarly, used the Glasgow Coma Score (GCS) of 8 or lower, or did not provide a clear definition.” [1] In our opinion, this means that there is no clear definition of coma. If a GCS of 8 or lower is the definition of coma in post cardiac arrest patients, post cardiac arrest patients with GCS: E2VTM5 and GCS: E1VTM5 would require MTH. Likewise, there is no clear definition of coma in the ERC guideline. We think that there is room for discussion about MTH for post cardiac arrest cases with a GCS motor response score of 5.

**Table 4:** Definition of coma

Author	Period	Definition of coma
Mckean et al. [5]	2009	GCS ≤ 8 and E1 and M ≤ 5
Testori et al. [6]	2011	GCS ≤ 8
Hörburger et al. [7]	2012	GCS < 8
Nielsen et al. [8], [9]	2013	GCS < 8
Hachimi-Idrissi et al. [10]	2005	GCS < 7
Shah et al. [12]	2011	GCS E1 or M1
HACA study group [14]	2002	GCS M ≤ 5
Bernard et al. [13]	2002	None
Knafelj et al. [15]	2007	None
Derwall et al. [16]	2009	None
Petrovic et al. [17]	2011	None

*GCS* Glasgow Coma Scale, *E* eye opening score, *M* motor response score

There were several reasons why we decided on GCS M5 as the optimal motor score threshold for MTH. Firstly, the result of the CHAID analysis indicated that an optimal GCS motor score threshold for MTH was GCS M5. Secondly, the percentage of patients with a good recovery in the GCS M5 and M6 groups was around 100 %, and for the patients with a bad recovery in the GCS M5 and M6 groups, it was difficult to believe that implementing MTH would have improved the CPC at 30 days after hospital admission. Thirdly, the ORs of GCS M5 and GCS M6 for good recovery were significantly higher, whilst the percentages of patients with a good recovery in the GCS M4, M3, M2, and M1 were lower than 60 %. Fourthly, MTH improved CPC at 30 days after hospital admission in the GCS M2 and M4 groups.

As for the reason why no significant difference on CPC at 30 days after hospital admission between with MTH and without MTH was observed in the GCS M1 and M3 groups, though the patients treated with MTH had a significantly better neurological outcome at 30 days after hospital admission in the GCS M2 and GCS M4 groups, we postulated that the GCS M1 group included patients whose neurological outcome was poor regardless of whether the patients were treated with MTH, and the GCS M3 group had a small number of patients. Because the OR of MTH for good recovery was 2.901 (*P* = 0.002), there may be patients whose neurological outcome was good because of MTH in the GCS M1 group.

From the above, because there are post cardiac arrest patients who receive a beneficial effect from MTH among those with a GCS motor response score of 4 or lower, we suggest that the definition regarding the degree of unconsciousness for which MTH is indicated in post cardiac arrest patients is a GCS motor response score of 4 or lower. Furthermore, when discussing the efficacy of MTH, “the percentage of patients with a GCS motor response score of 5 or higher” should be clarified.

In this study, patients with a GCS motor score of 4 or lower had relatively lower percentage of good recovery than earlier trials [13, 14]. The reason was that we included patients with out-of-hospital arrests of presumed non-cardiac cause, non-shockable rhythms and we had fewer exclusion criteria. Another published study [9] involving patients with cardiac arrest who were admitted to the ICU demonstrated baseline characteristics and mortality that are in keeping with our findings.

Regarding the limitations of this study, the number of patients evaluated was insufficient to make any definitive conclusions. This was a retrospective study at a single institute, and there were some differences in the patient background between patients who were treated with MTH and patients who were treated without MTH. Moreover, the presence of higher brain dysfunction was not evaluated in CPC1 patients at 30 days after hospital admission, so from the standpoint of preventing higher brain dysfunction, the efficacy of MTH in patients classified according to a GCS motor response score of 5 or higher cannot be discussed. We hope this study will be seen as a pilot study leading to a prospective, randomized, multi-center study in the future.

## Conclusions

MTH may be unnecessary in patients with a GCS motor response score of 5 or higher. Consequently, because there are post cardiac arrest patients who receive a beneficial effect from MTH among patients with a GCS motor response score of 4 or lower, the degree of unconsciousness in out-of-hospital cardiac arrest patients for which MTH is indicated may be limited to patients with a GCS motor response score of 4 or lower. Furthermore, when discussing the efficacy of MTH, “the percentage of patients with a GCS motor response score of 5 or higher” should be clarified.

### Key messages

Out-of-hospital cardiac arrest patients with a GCS motor response score of 5 or higher showed good neurological outcomes, whether or not MTH was performed.

MTH for post cardiac arrest syndrome may be performed in patients with a GCS motor response score of 4 or lower.

## Abbreviations

CPC: Cerebral Performance Category
GCS: Glasgow Coma Scale
GCS M1: patients classified with a GCS motor response score of 1
GCS M1–4: patients classified with a GCS motor response score of 4 or lower
GCS M2: patients classified with a GCS motor response score of 2
GCS M2-4: patients classified with a GCS motor response score from 2 to 4
GCS M3: patients classified with a GCS motor response score of 3
GCS M4: patients classified with a GCS motor response score of 4
GCS M5: patients classified with a GCS motor response score of 5
GCS M5–6: patients classified with a GCS motor response score of 5 or higher
GCS M6: patients classified with a GCS motor response score of 6
ICU: intensive care unit
MTH: mild therapeutic hypothermia
PCPS: percutaneous cardio-pulmonary support
ROSC: return of spontaneous circulation
VF: ventricular fibrillation
VT: ventricular tachycardia

## References

[1] Peberdy M, Callaway C, Neumar R, Geocadin R, Zimmerman J, Donnino M, et al. American Heart Association Guidelines for Cardiopulmonary Resuscitation and Emergency Cardiovascular Care Part 9 post-cardiac arrest care. Circulation. 2010;122:S768–S786. 10.1161/CIRCULATIONAHA.110.971002 20956225

[2] Erb JL, Hravnak M, Rittenberger JC (2012). Therapeutic hypothermia after cardiac arrest. Am J Nurs.

[3] Teasdale G, Maas A, Lecky F, Manley G, Stocchetti N, Murray G (2014). The Glasgow Coma Scale at 40 years: standing the test of time. Lancet Neurol.

[4] Phelps R, Dumas F, Maynard C, Silver J, Rea T (2013). Cerebral performance category and long-term prognosis following out-of-hospital cardiac arrest. Crit Care Med.

[5] McKean S (2009). Induced moderate hypothermia after cardiac arrest. AACN Adv Crit Care.

[6] Testori C, Sterz F, Behringer W, Haugk M, Uray T, Zeiner A (2011). Mild therapeutic hypothermia is associated with favourable outcome in patients after cardiac arrest with non-shockable rhythms. Resuscitation.

[7] Testori C, Sterz F, Behringer W, Haugk M, Uray T, Zeiner A (2012). Mild therapeutic hypothermia improves outcomes compared with normothermia in cardiac-arrest patients. A retrospective chart review. Crit Care Med.

[8] Nielsen N, Wetterslev J, al-Subaie N, Andersson B, Bro-Jeppesen J, Bishop G, et al. Target temperature management after out-of-hospital cardiac arrest—a randomized, parallel group, assessor-blinded clinical trial—rationale and design. Am Heart J. 2012;163:541–8. 10.1016/j.ahj.2012.01.013 22520518

[9] Nielsen N, Wetterslev J, Cronberg T, Erlinge D, Gasche Y, Hassager C, et al. Targeted temperature management at 33 °C versus 36 °C after cardiac arrest. N Engl J Med. 2013;369:2197–206. 10.1056/NEJMoa1310519 24237006

[10] Hachimi-Idrissi S, Corne L, Ebinger G, Michotte Y, Huyghens L (2001). Mild hypothermia induced by a helmet device: a clinical feasibility study. Resuscitation.

[11] Hachimi-Idrissi S, Zizi M, Nguyen DN, Schiettecate J, Ebinger G, Michotte Y, et al. The evolution of serum astroglial S-100 β protein in patients with cardiac arrest treated with mild hypothermia. Resuscitation. 2005;64:187–92. 10.1016/j.resuscitation.2004.08.008 15680528

[12] Shah MP, Zimmerman L, Bullard J, Yenari MA (2011). Therapeutic hypothermia after cardiac arrest: experience at an academically affiliated community-based veterans affairs medical center. Stroke Res Treat.

[13] Bernard SA, Gray TW, Buist MD, Jones BM, Silvester W, Gutteridge G (2002). Treatment of comatose survivors of out-of-hospital cardiac arrest with induced hypothermia. N Engl J Med.

[14] Holzer M, Cerchiari E, Roine R, Sterz F, Eisenburger P, Havel C, et al. Mild therapeutic hypothermia to improve the neurologic outcome after cardiac arrest. N Engl J Med. 2002;346:549–56. 10.1056/NEJMoa012689 11856793

[15] Knafelj R, Radsel P, Ploj T, Noc M (2007). Primary percutaneous coronary intervention and mild induced hypothermia in comatose survivors of ventricular fibrillation with ST-elevation acute myocardial infarction. Resuscitation.

[16] Derwall M, Stoppe C, Brücken D, Rossaint R, Fries M (2009). Change in S-100 protein serum levels in survivors of out-of-hospital cardiac arrest treated with mild therapeutic hypothermia: a prospective, observational study. Crit Care.

[17] Petrović M, Panić G, Jovelić A, Canji T, Srdanović I, Popov T (2011). Therapeutic hypothermia and neurological outcome after cardiac arrest. Vojnosanit Pregled.

